# Design and Verification of Deep Submergence Rescue Vehicle Motion Control System

**DOI:** 10.3390/s23156772

**Published:** 2023-07-28

**Authors:** Chunmeng Jiang, Hongrui Zhang, Lei Wan, Jinhua Lv, Jianguo Wang, Jian Tang, Gongxing Wu, Bin He

**Affiliations:** 1 Wuhan Institute of Shipbuilding Technology, Wuhan 430050, China; 2020017@mail.wspc.edu.cn (C.J.);; 2 School of Naval Engineering, Harbin Engineering University, Harbin 150001, China; 3 China Ship Development and Design Center, Wuhan 430064, China; 4 College of Ocean Science and Engineering, Shanghai Maritime University, Shanghai 201306, China; 5 Wuhan Second Ship Design and Research Institute, Wuhan 430205, China

**Keywords:** DSRV, motion control system, sparse filtering, thrust allocation, automatic inclination control, ballast tank regulation

## Abstract

A six degree-of-freedom (DOF) motion control system for docking with a deep submergence rescue vehicle (DSRV) test platform was the focus of this study. The existing control methods can meet the general requirements of underwater operations, but the complex structures or multiple parameters of some methods have prevented them from widespread use. The majority of the existing methods assume the heeling effect to be negligible and ignore it, achieving motion control in only four or five DOFs. In view of the demanding requirements regarding positions and inclinations in six DOFs during the docking process, the software and hardware architectures of the DSRV platform were constructed, and then sparse filtering technology was introduced for data smoothing. Based on the adaptive control strategy and with a consideration of residual static loads, an improved S-plane control method was developed. By converting the force (moment) calculated by the controller to the body coordinate system, the complexity of thrust allocation was effectively reduced, and the challenge of thrust allocation in the case of a high inclination during dynamic positioning was solved accordingly. The automatic control of the trimming angle and heeling angle was realized with the linkage system of the ballast tank and pump valve. A PID method based on an intelligent integral was proposed, which not only dealt with the integral “saturation” problem, but also reduced the steady-state error and overshooting. Water pool experiments and sea trials were carried out in the presence of water currents for six-DOF motion control. The responsiveness and precision of the control system were verified by the pool experiment and sea trial results and could meet the control requirements in engineering practice. The reliability and operational stability of the proposed control system were also verified in a long-distance cruise.

## 1. Introduction

Significant progress has been made in underwater vehicle technologies along with the booming development in big data [1], data mining [2], computer simulation [3], intelligent control [4], intelligent optimization [5], virtual reality [6], and artificial intelligence [7]. Underwater vehicles are playing an increasingly important part in workplaces where equipment and divers face access difficulties such as detection and salvage [8], dam detection [9], pipeline maintenance [10], cable servicing and laying [11,12], and marine resource development [13]. With their widespread applications in both civil and military fields [14,15,16,17], underwater vehicles have become an important tool in underwater operations. Underwater vehicles are equipped with different sensors and devices for different tasks, so reliable performance has been the main goal of controller design, especially for the highly coupled nonlinear systems [18,19] of underwater vehicles.

In order to meet the varying demands for the motion control of autonomous underwater vehicles (AUVs) in different operating conditions, studies and discussions have been conducted on different control methods for decades. Jin Sangrok et al. [20] designed a proportional derivative (PD) controller to deal with AUV hovering control in six DOFs. The simulation experiments’ results proved the effectiveness of the PD controller. The results of enhanced experiments further verified the advantage of the proposed method in long-term control. Harsh Goud et al. [21] worked on a classic proportional integral derivative (PID) controller. The constraints in the form of fixed parameters were deficient, so a new method based on swarm optimization was adopted to calculate the PID parameters. Particle swarm optimization was used to regulate the PID parameters. Simulation experiments were carried out to verify the advantages of the proposed PID method over PD control. Bingul Zafer et al. [22] focused on model-independent trajectory tracking control. A hybrid controller was put forward by combining intelligent PID and PD feed-forward controllers for the purpose of a higher anti-interference effect and compensation for initial tracking errors. Numerical simulation experiments under different interference conditions showed the satisfactory trajectory tracking performance and anti-interference effect of the hybrid controller. Based on existing kinematics and dynamics models, a fuzzy controller [23] was developed for AUV path tracking control. Particle swarm optimization was used to determine the membership functions. The effectiveness and feasibility of the fuzzy controller were verified by the simulation results. Keymasi Khalaji Ali et al. [24] proposed a finite-time sliding-mode controller for underwater vehicle robust control in 3D space. In contrast to two-dimensional motion control, a new steady-state AUV control algorithm was designed. The stability of the closed-loop system was analyzed via the Lyapunov stability theory. The robust method could reduce the influence of external interference. The proposed method was compared with the sliding-mode controller in terms of performance and stability. The numerical simulation results proved the effectiveness of the proposed method. Hernandez-Sanchez Alejandra et al. [25] concentrated on a novel robust control strategy for underwater vehicles. The proposal was an integral sliding-mode controller based on state variable gains to deal with the tracking of a predefined reference trajectory. The optimization of the penalty functions did not require complete knowledge of AUV dynamics. Contrastive simulation experiments were conducted between the proposed strategy and a PID controller. The experiments proved that the trajectory tracking results of the proposed strategy were better than those of the PID controller. TabatabaeeNasab Fahimeh S. et al. [26] conducted a study on kinematic and dynamic models to realize trajectory tracking control in the case of failures on under-actuated AUVs. A fault-tolerant strategy was adopted to ensure stable operation. An adaptive rule was taken as the on-line estimation to compensate for the partial malfunctions of the AUV. The proposed method was compared with the classic PID and fuzzy control in simulation experiments. A robust model predictive control method based on the active elimination of interference was developed [27]. Simulation experiments proved the advantages of the proposed model prediction controller over classic model predictive control in terms of reference trajectory and resistance to external interference. Ru et al. [28] studied the task distribution of multiple AUVs. A fast graph positioning network was designed to solve the task distribution of detecting/communicating AUV clusters with the aim of improving the task distribution efficiency. The effectiveness and higher processing efficiency of the proposed network was verified in a number of simulation experiments. Wang et al. [29] came up with an adaptive fuzzy controller based on a fuzzy performance observer and fuzzy interference observer, together with a novel dead zone pre-compensator. The on-line adaptive fuzzy logic system was used to evaluate the unknown components in the inertial matrix. The H∞ fuzzy control technique was applied to the reduction of the estimated external interference deviation. Simulation experiments verified the effectiveness of the proposed controller in tracking control. Pham DucAnh et al. [30] designed a fuzzy neural network as an AUV controller. The neural network was combined with fuzzy logic to improve the efficiency of trajectory tracking. Without the need for the regulation of the gain in PID control, the proposed network showed a faster response and higher resistance to external interference. The network can be applied to AUVs of different weights and dimensions. An adaptive fuzzy nonlinear PID controller was put forward [31] to counter the external interference with underwater vehicles. The contrastive simulation experiments with the classic PID controller and the adaptive fuzzy PID controller verified the advantages of the proposed method. Sedghi Fatemeh et al. [32] worked on AUV finite-time trajectory tracking control in six DOFs. Based on the back-stepping method, a finite-time control input was proposed and designed. The artificial neural network and the finite-time adaptive law were used to approximate the nonlinear dynamic characteristics of the AUV. The finite-time instruction filtering method with compensation was also discussed. The aforesaid adaptive finite-time control method was proved to be effective in simulation experiments. To some extent, the above control methods can meet the requirements of control accuracy and some have been applied in engineering practice. However, some of these methods are designed based on complex structures [33,34,35,36,37], some having multiple control parameters needing regulation [38,39,40] and some highly dependent on personal experience and field trials in parameter determination [41,42,43], which have restrained them from widespread use in practice. Moreover, the methods proposed in the existing studies are mostly verified in the simulation experiments instead of field trials. 

Among these control methods, the classic S-plane method has been proven to be an effective and practical method in extensive engineering practices. Combining the PD control structure with the fuzzy logic, the classic S-plane method has been verified in pool experiments and sea trials and can well meet the accuracy requirements in routine underwater operations [44,45]. Nevertheless, the impact of variable static loads is not taken into consideration by the classic S-plane method, which will degrade the control accuracy, especially in operations with high demands on control accuracy.

Relying on underwater vehicle technologies, deep submergence rescue vehicles (DSRV) are needed and expected to complete high-precision docking operations with the wrecked object in six DOFs [46,47,48,49,50]. More flexible manipulation and intelligent control technologies are required. The docking process demands accurate positioning together with precise control over the trimming angle and heeling angle. Therefore, the inclination control ability and operational stability of dynamic positioning under high inclination are of great importance to successful docking.

The main contributions of this paper include a filtering method for sensor data, thrust allocation strategies considering the impacts of the heeling angle, trimming angle and the realization of AUV control in six DOFs. The pump-valve linkage system is designed to realize control in the pitch and roll directions. With consideration of the impact of static loads, improvements are made to the classic S-plane method for better control performance. 

The article is organized as follows. The second section provides a description of the test platform, including the structure framework, hardware and software architectures. The third section focuses on the controller design, including sensor data filtering, an improved S-plane method considering the residual static loads, a thrust distribution strategy and inclination calculation. In the fourth part, contrastive simulation experiments are carried out between the proposed method and the classic S-plane control method. The fifth and sixth sections provide the results and analysis of pool experiments and sea trials.

## 2. DSRV Platform

### 2.1. Platform Framework

**Figure 1:**
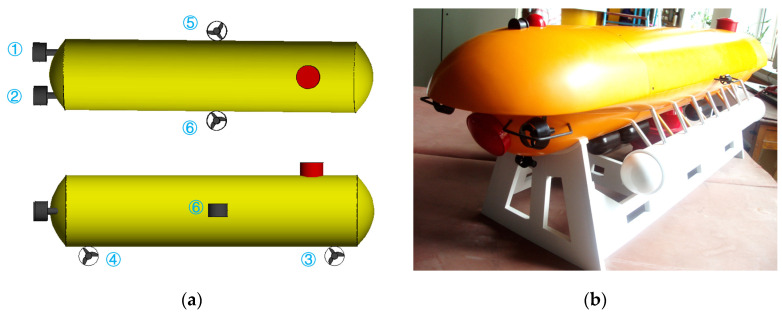
Profile of the DSRV. (**a**) Schematic diagram; (**b**) scaled model of the actual system.

The schematic diagram of the DSRV platform is shown in Figure 1a. It realizes functions of motion control, planning, navigation, self-rescue, surface monitoring, etc. [51]. As an intelligent vehicle, its planning system, motion control system, navigation system and self-rescue system are embedded in the framework of the platform. The platform is monitored through the surface monitoring system. The performance in motion control is given the top priority [52].

The DSRV platform weighs approximately 1.6 t, 4.5 m long, 1.2 m wide and 1.3 m tall. The main body is designed to be streamlined in a double hull structure, namely the pressure hull and the outer shell. The pressure hull provides a reliable pressurized and watertight context for electronic control and batteries. It is made of aluminum alloy and composed of the head, the parallel main body and the tail, which are connected with sealing rings. The outer shell is made of glass-reinforced plastics and designed in a streamlined shape to counter water resistance. The entire platform is equipped to be of slightly positive buoyancy. 

The platform is driven by thrusters and the pump-valve linkage system. There are six thrusters and accessories, including the main thrusters, the side thrusters and the vertical thrusters. The switching frequency of the thrusters is 20 kHz. Among the six regulating water tanks, four are used for the heeling control designed outside the pressure hull and two are used for the trimming control designed at the bow and the stern, respectively.

### 2.2. Hardware Architecture

**Figure 2:**
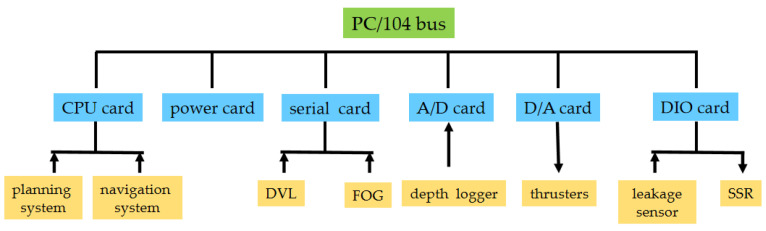
Hardware architecture of the platform.

As shown in Figure 2, the hardware architecture is based on the PC104 embedded system with VxWorks as the real-time operating system [53]. The central processing unit (CPU) card, power card, A/D card, D/A card, digital input and output (DIO) card and serial port card [45] are integrated in the architecture. The motion sensors equipped with the DSRV include the Fiber-Optic Gyroscope (FOG), Doppler Velocity Log (DVL) and depth logger. The FOG provides a heading resolution of 0.01°, together with a dynamic accuracy of ±0.2° in heading control and 0.01° in roll/pitch control. The DVL provides an accuracy of (±0.2%±0.1) cm/s. And the depth logger provides a resolution of 0.001% with an accuracy of 0.01% in distance. The feasibility and effectiveness of the hardware architecture have been verified in a number of lake and sea trials on underwater vehicles of different models.

The CPU card guarantees the operating environment. The serial port card collects data from the FOG and DVL. The depth data are collected by the A/D card. The D/A card transmits voltage instructions to the thrusters. The DIO card receives data from the leakage sensor and sends digital information to the pumps and valves. The power card provides power supply to all interface cards. Network and serial ports are responsible for communication [54]. The cards in the embedded system communicate via the PC104 bus. User datagram protocol (UDP) is adopted for communication with the planning system, transmission control protocol (TCP) for communication with the navigation system and RS232 protocol for communication with the sensors.

### 2.3. Software Architecture

The motion control system is modularly designed for parallel development as well as higher commonality, portability and expansibility [55,56,57]. The software architecture covers functions of data acquisition and decoding, data filtering processing, control algorithm calculation, thrusting force allocation, inclination calculation, instruction transmission and data storage of the thrusters, pumps and valves. The software is developed with the C programming language based on the watchdog mechanism and provides multi-task parallel processing. Sensor data are acquired via serial ports based on the event-driven mechanism with “first in first out” priority.

**Figure 3:**
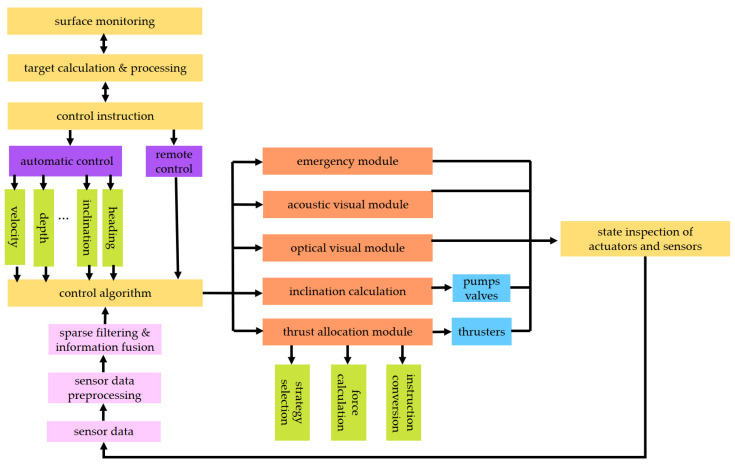
Workflow of the DSRV platform.

The workflow of the DSRV platform is demonstrated in Figure 3. To start with, the surface monitoring module issues the target instructions via radio or underwater acoustic communication. The target calculation and processing module receives the instructions and has the targets classified. Then, the control instruction module has the control targets initialized, including the setting of the motion control model, the type of motion control and the initial parameters. The above data are then sent to the control algorithm module, together with the sensor data that have been collected and filtered. As the most essential part of the software architecture, the control algorithm module can respond to remote control and automatic control requirements. In automatic control, the control algorithm computes the target instruction and sends the calculation results to the thrust allocation module and the inclination calculation module. Then, the thrusting force expected from each thruster and on–off instructions expected from the pump-valve linkage system are obtained. The thrusting forces and on-off instructions are then converted into analog voltage as well as the opening act and duration of the pumps and valves, which will drive the DSRV toward the control target.

The information among modules is transmitted as follows. The surface monitoring module transmits binary target instructions to the module of target calculation and processing by way of radio or underwater communication, including the control target, the control mode or emergency-triggering condition. Then, the target instructions in decimal are transmitted to the control instruction module. The control instruction module transmits initialized control parameter data to the control algorithm, which accordingly sends digital thrusting (torque) data to the thrust allocation module as well as digital data of the heeling/trimming angle to the inclination calculation module at the same time. The thrusters receive analog voltage instructions from the thrust allocation module while the pump-valve linkage system receives the on–off instructions from the inclination calculation module.

## 3. Control System Design

### 3.1. Sparse Filtering Method

The platform for different operations requires high accuracy of the navigation sensor [58]. According to the characteristics of DVL, the filtering technology based on sparse representation is adopted to reduce noise interference [59] and improve data accuracy.

#### 3.1.1. DVL Data in Sparse Representation

The variable s is a discrete data vector that represents the velocity information measured by the DVL. It is composed of groups of velocity in surge, sway and heave directions. The variable s is discrete real outputs of DVL with finite length. $s\in {ℜ}^{3\times N_{s}}$ and it is $N_{S}$×3 in dimension. All the elements in ${ℜ}^{3\times N_{s}}$ can be expressed by a unit vector $\left\{b_{1},b_{2},b_{3},\cdots b_{N_{s}}\right\}$ in dimension of $N_{S}$× 3. The variable $b_{i}$ is pairwise orthogonal. The variable s is expressed as in Equation (1), where the variable $\beta_{i}$ is a column vector. The variable $\beta_{i}$ means sparse representation coefficients and they constitute $\beta ={\left[\beta_{1},\beta_{2},\beta_{3}\cdots \beta_{N_{S}}\right]}^{T}$.

(1) $$s=\sum_{i=1}^{N_{S}}\beta_{i}b_{i}$$

With a known or trained dictionary B, sparse representation can be used to de-noise the data and then sparse reconstruction is conducted to have the data recovered via $s=B\hat{\beta }$. The variable $\hat{\beta }$ is expected to satisfy Equations (2) and (3), where $\hat{\beta }$ is the estimation of β. The variable ${\left\|\beta \right\|}_{1}$ is the statistics of the sum of absolute values of all elements in $\beta_{i}$. The variable $\delta_{n}$ is determined by the standard deviation of the data noise.

(2) $$\hat{\beta }=\min{\left\|\beta \right\|}_{1}$$

(3) $$s.t.{\left\|B\beta -s\right\|}_{2}^{2}\leq \delta_{n}$$

Equations (2) and (3) are converted into the optimization expression as shown in Equation (4), where $L_{G}$ is the Lagrange multiplier.

(4) $$\hat{\beta }=\underset{\beta }{\arg\min}\left\{{\left\|\beta \right\|}_{1}+L_{G}{\left\|s-B\beta \right\|}_{2}^{2}\right\}$$

#### 3.1.2. Filtering Model

**Figure 4:**
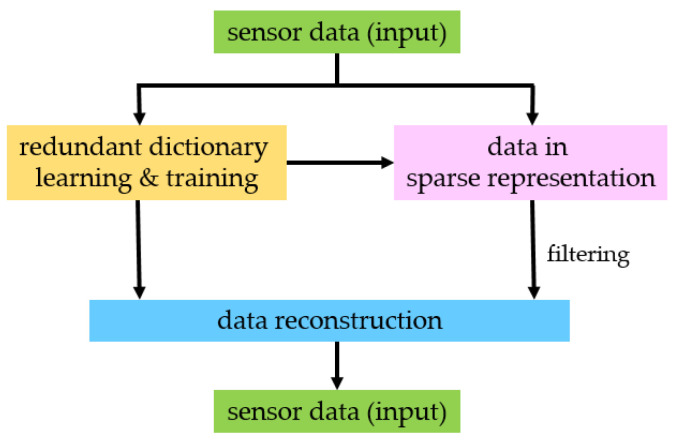
Sparse representation filtering model.

A real-time filtering method based on sparse representation is adopted. The established filtering model consists of the unit of dictionary learning and training and the unit of filtering. As shown in Figure 4, the unit of filtering completes sparse representation and sparse reconstruction of data. In the unit of dictionary learning and training, the dictionary is constructed with learning and training via the K-singular value decomposition (K-SVD) algorithm [60,61]. The raw data are processed by sparse representation and reconstruction in accordance with the dictionary to realize data filtering.

#### 3.1.3. Simulation Experiments of Data Filtering

Under different thrusting forces and interference conditions, simulation experiments on DVL data filtering were carried out to verify the feasibility and effectiveness of the aforesaid method. 

The dictionary was set to a size of 3 × 8 and only 1–2 dictionary atoms were engaged in data reconstruction during the learning and training process. For a more precise representation of the data, the samples with a size of 3 × 200 were selected. When the dictionary was trained, the orthogonal matching tracking method [62] was used to solve Equations (2) and (3) to realize sparse representation.

**Figure 5:**
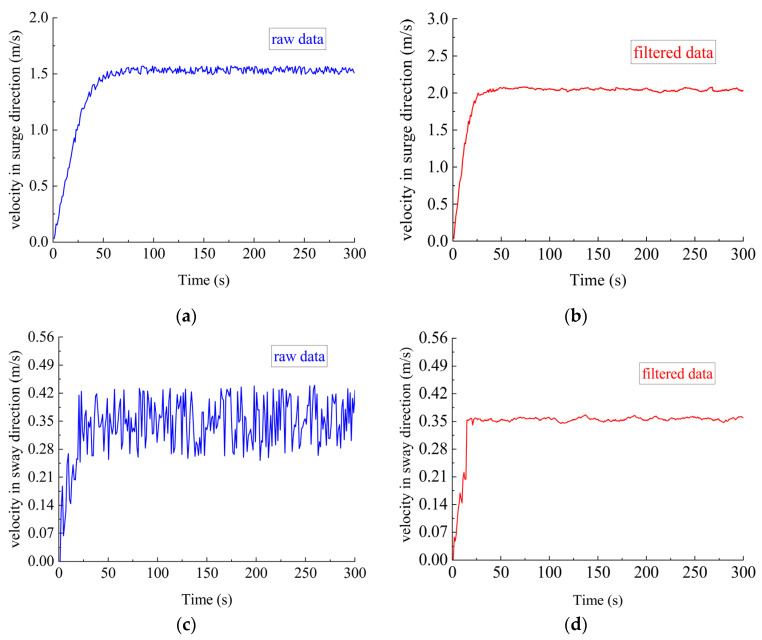
Velocity data before and after filtering (simulation Scenario II). (**a**) Velocity in surge direction before filtering; (**b**) velocity in surge direction after filtering; (**c**) velocity in sway direction before filtering; (**d**) velocity in sway direction after filtering.

Simulation Scenario I. The DSRV was stationary on the water surface. The desired velocity was 1.5 m/s in the surge direction and 0.3 m/s in the sway direction. The water current was 0.25 m/s with a flow direction of 15°. The DVL error was 0.1 m/s and followed (0, 0.1) Gaussian distribution. The filtering results are shown in Figure 5.

As shown in Figure 5, although DVL data experienced more challenging noise interference from the water current conditions, the over-complete redundant dictionary still filtered the noise interference well and improved the output accuracy.

#### 3.1.4. Data Filtering of Sea Trials

To further verify the effectiveness of the filtering method, data filtering in sea trials was conducted with the same dictionary as used in the simulation experiments. The initial state was stationary. The desired velocity was 1.8 m/s in the surge direction and 0.28 m/s in the sway direction. The site of the sea trial was subject to sea current interference.

**Figure 6:**
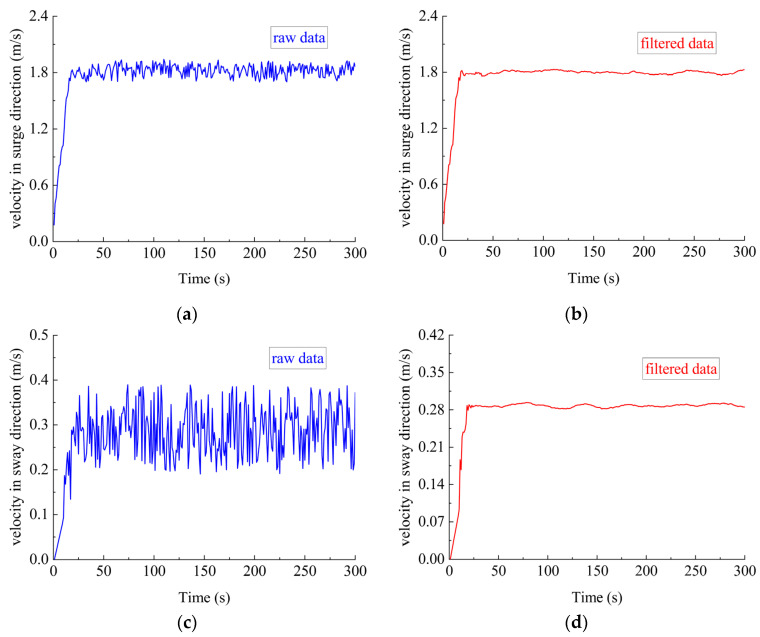
Velocity data before and after filtering (sea trial). (**a**) Velocity in surge direction before filtering; (**b**) velocity in surge direction after filtering; (**c**) velocity in sway direction before filtering; (**d**) velocity in sway direction after filtering.

The curve of the filtered data was smooth, as shown in Figure 6, with the frequent noises well eliminated. The results proved that the sparse representation can improve the accuracy of DVL outputs and provide the motion control system with high-precision data inputs.

### 3.2. Classic S-Plane Control Algorithm

The classic S-plane controller based on a mathematical model [44,45] is shown in Equation (5).

(5) $$\left\{\begin{array}{l} O=\frac{2.0}{1+\exp(-k_{e}e-k_{\dot{e}}\dot{e})}-1\\ T_{c}{=T}_{\max}O \end{array}\right.$$

In Equation (5), O is the normalized control output. exp() stands for the exponential function. e the normalized deviation and $\dot{e}$ the variation rate of e. $e={\left(u_{d}-{u\;y}_{d}-{y\;z}_{d}-z\;\phi_{d}-\phi {\;\theta }_{d}-{\theta \;\psi }_{d}-\psi \right)}^{T}$, $\dot{e}={\left(a_{u}\;v\;w\;p\;q\;r\right)}^{T}$. $k_{e}$ and $k_{\dot{e}}$ are, respectively, the control parameters of e and $\dot{e}$. $T_{c}$ is the expected thrusting force (or torque) calculated by the control method. $T_{\max}$ is the maximum thrusting force (or torque) that the AUV can provide [45].

### 3.3. Improved S-Plane Control Algorithm

Although the classic S-plane method has been recognized as a practical approach that can well meet the requirements of control accuracy in regular underwater operations [44], it does not consider the situational static load. However, DSRVs carry different operation devices and testing equipment as required in different tasks, which will certainly produce situational static loads. The lack of consideration of such static loads will accordingly affect the control results. In order to counter the impact of the situational static load and improve the control accuracy, an improved S-plane control method based on an adaptive strategy is proposed with full consideration of the situational static load. The method is described in Equation (6).

(6) $$\left\{\begin{array}{l} u_{i}=2/(1+\exp(-k_{i1}e_{i}-k_{i2}{\dot{e}}_{i}))-1+\Delta u_{i}\\ f_{i}=K_{i}u_{i}+u_{s} \end{array}\right.$$

In Equation (6), the variable $u_{i}$ is the normalized controller output meaning the thrusting force or moment required in the ith DOF. The variables i = 1, 2, 3, and 6 refer to the directions of surge, sway, heave and yaw. The variables $e_{i}$ and ${\dot{e}}_{i}$ are the normalized control inputs meaning the deviation and the deviation change rate in the ith DOF. The variables $k_{i1}$ and $k_{i2}$ are the regulating parameters of $e_{i}$ and ${\dot{e}}_{i}$. The regulation of $k_{i1}$ and $k_{i2}$ determines the overshoot size and convergence of the control target in the ith DOF. The variable $\Delta u_{i}$ is the fixed interference force during a certain period calculated by the adaptive method. The variable $f_{i}$ is the thrusting force or moment required in the ith DOF. The variable $K_{i}$ is the maximum thrusting force or moment that can be provided in the ith DOF. The variable $u_{s}$ is the integral term eliminating the fixed deviation with the consideration of the static load in the ith DOF.

### 3.4. Thrust Allocation Strategy

**Figure 7:**
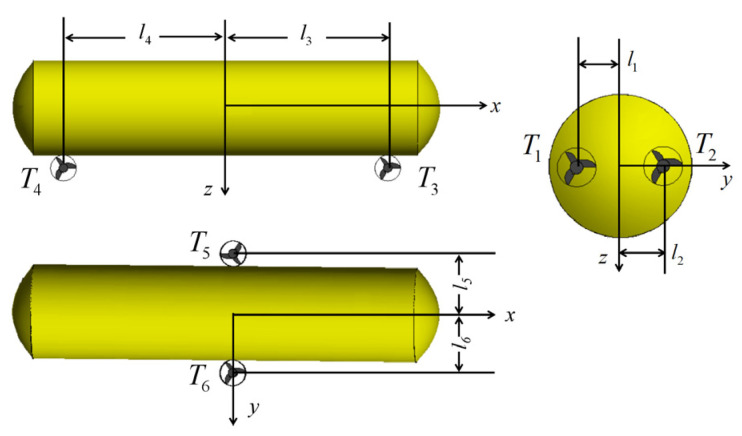
Layout of platform thrusters.

It can be seen from Figure 1 and Figure 7 that the DSRV is equipped with two main thrusters, two side thrusters and two vertical thrusters. As mentioned above, the six thrusters realize control of the heading angle and movement in the surge, sway and heave directions. The pump-valve linkage system realizes control of the heeling angle and the trimming angle. The thrusters are arranged as shown in Figure 7. Variable $T_{1}$ and $T_{2}$ are the thrusting forces of the left main thruster and the right main thruster, respectively, with $l_{1}$ and $l_{2}$ being the corresponding arms of force. The variables $T_{3}$ and $T_{4}$ are the thrusting forces of the side thrusters at the bow and the stern, respectively, with $l_{3}$ and $l_{4}$ being the corresponding arms of force. The variables $T_{5}$ and $T_{6}$ are the thrusting forces of the left vertical thruster and the right vertical thruster, respectively, with $l_{5}$ and $l_{6}$ being the corresponding arms of force.

The platform provides services mainly for position and attitude control as well as long-distance cruise. Most of the operation tasks are navigation without inclination. Significant trimming angles and heeling angles are required only in operations of docking or dynamic positioning with inclination. The thrust allocation strategy is explained in accordance with the above two situations.

#### 3.4.1. Thrust Allocation Strategy for Control without Inclination

In the case of control without inclination, the fixed coordinate system is considered in the same direction of axes as the movement coordinate system. The forces (moments) required in the four DOFs and those provided by the six thrusters follow the relation as shown in Equation (7). The matrix ${\left[\begin{array}{cccc} X_{0} & Y_{0} & Z_{0} & N_{0} \end{array}\right]}^{T}$ is the force (moment) in the fixed coordinate system and ${\left[\begin{array}{cccc} X & Y & Z & N \end{array}\right]}^{T}$ is the force (moment) in the movement coordinate system.

(7) $$\left[\begin{array}{c} X_{0}\\ Y_{0}\\ Z_{0}\\ N_{0} \end{array}\right]=\left[\begin{array}{c} X\\ Y\\ Z\\ N \end{array}\right]=\left[\begin{array}{c} 1\\ 0\\ 0\\ l_{1} \end{array}\begin{array}{c} 1\\ 0\\ 0\\ -l_{2} \end{array}\begin{array}{c} 0\\ 1\\ 0\\ l_{3} \end{array}\begin{array}{c} 0\\ 1\\ 0\\ -l_{4} \end{array}\begin{array}{c} 0\\ 0\\ 1\\ 0 \end{array}\begin{array}{c} 0\\ 0\\ 1\\ 0 \end{array}\right]\left[\begin{array}{c} T_{1}\\ T_{2}\\ T_{3}\\ T_{4}\\ T_{5}\\ T_{6} \end{array}\right]$$

It can be seen from Equation (7) that the control of thrusters is redundant. Theoretically, the pseudo-inverse technique can be adopted to obtain the thrusting force. In practice, however, for simplified calculations, constraint conditions are introduced according to the characteristics of actual tasks. With the same spacing with the mid-longitudinal profile of the platform, the two main thrusters of the platform are responsible for movement in the surge direction only.

(8) $$\left\{\begin{array}{l} T_{1}l_{1}-T_{2}l_{2}=0\\ l_{1}=l_{2} \end{array}\right.$$

The vertical thrusters are responsible for control in the vertical direction only and have the same spacing with the mid-longitudinal profile of the platform.

(9) $$\left\{\begin{array}{l} T_{5}l_{5}-T_{6}l_{6}=0\\ l_{5}=l_{6} \end{array}\right.$$

Based on Equations (7)–(9), the thrusting force required upon each thruster is calculated as in Equation (10).

(10) $$\left\{\begin{array}{l} T_{1}=X_{0}/2\\ T_{2}=X_{0}/2\\ T_{3}=(Y_{0}l_{4}-N_{0})/(l_{4}-l_{3})\\ T_{4}=(N_{0}-Y_{0}l_{3})/(l_{4}-l_{3})\\ T_{5}=Z_{0}/2\\ T_{6}=Z_{0}/2 \end{array}\right.$$

It is assumed that the maximum thrusting force provided by the main thrusters is $T_{X\max}$, provided by the side thrusters is $T_{Y\max}$, and provided by the vertical thrusters is $T_{Z\max}$. If the calculated forces provided by the main thrusters (namely $T_{1}$ and $T_{2}$) and provided by the vertical thrusters (namely $T_{5}$ and $T_{6}$) are greater than the maximum thrusting force provided by the thrusters, they follow the rules in Equation (11).

(11) $$\left\{\begin{array}{l} \left|T_{1}\right|=T_{X\max}\\ \left|T_{2}\right|=T_{X\max}\\ \left|T_{5}\right|=T_{Z\max}\\ \left|T_{6}\right|=T_{Z\max} \end{array}\right.$$

The thrusting forces from the side thrusters (namely $T_{3}$ and $T_{4}$) are dealt with differently in the following two situations.

(a) There is only one calculated thrusting force that is greater than $T_{Y\max}$. It is assumed that $\left|T_{4}\right|<T_{Y\max}<\left|T_{3}\right|$. The side thrusters are responsible for control in the sway and yaw directions. In line with the dynamic characteristics of the platform and task requirements, the thrust allocation strategy gives top priority to the heading control. Variable $T_{3}$ and $T_{4}$ are calculated as in Equation (12).

(12) $$\left\{\begin{array}{l} \left|T_{3}\right|=T_{Y\max}\\ \left|T_{4}\right|=\frac{T_{Y\max}l_{3}-N_{0}}{l_{4}} \end{array}\right.$$

(b) If the calculated thrusting forces upon the two side thrusters are greater than $T_{Y\max}$, $T_{3}$ and $T_{4}$ are calculated as in Equation (13).

(13) $$\left\{\begin{array}{l} \left|T_{3}\right|=T_{Y\max}\\ \left|T_{4}\right|=T_{Y\max} \end{array}\right.$$

#### 3.4.2. Thrust Allocation Strategy for Control with Inclination

When the platform is docked with the target, significant trimming and heeling angles are required. The thrusting forces provided by each thruster and needed in each DOF will accordingly become very complicated. For example, the vertical thrusters affect the movement not only in the vertical direction, but also the movement in the surge and sway directions. Therefore, it is necessary to study the thrust allocation strategy in case of DSRV control with inclination.

With a heeling angle of ϕ and a trimming angle of θ, the force (moment) required in each DOF in the movement coordinate system can be expressed by that in the fixed coordinate system as shown in Equation (14), where S stands for sine and C for cosine.

(14) $$\left[\begin{array}{l} X\\ Y\\ Z \end{array}\right]=\left[\begin{array}{ccc} C\theta & 0 & -S\phi \\ S\theta S\phi & C\phi & C\theta S\phi \\ S\theta C\phi & S\phi & C\theta C\phi \end{array}\right]\left[\begin{array}{l} X_{0}\\ Y_{0}\\ Z_{0} \end{array}\right]$$

(15) $$N_{0}=N(C\theta C\phi )$$

Based on Equations (7)–(9), (14) and (15), the thrusting force expected from each thruster is expressed as shown in Equation (16). If the calculated thrusting force is greater than the maximum thrusting force provided by the thrusters, the solution shall refer to that in control without inclination.

(16) $$\left\{\begin{array}{l} T_{1}=(P_{1})/2\\ T_{2}=(P_{1})/2\\ T_{3}=(P_{3}l_{4}-P_{4})/(l_{4}-l_{3})\\ T_{4}=(P_{4}-P_{3}l_{3})/(l_{4}-l_{3})\\ T_{5}=(P_{2})/2\\ T_{6}=(P_{2})/2 \end{array}\right.$$

According to Equation (15), $P_{1}$, $P_{2}$, $P_{3}$ and $P_{4}$ are defined in Equations (17)–(20).

(17) $$P_{1}=X_{0}C\theta -Z_{0}S\phi$$

(18) $$P_{2}=X_{0}S\theta C\phi +Y_{0}S\phi +Z_{0}C\theta C\phi$$

(19) $$P_{3}=X_{0}S\theta S\phi +Y_{0}C\phi +Z_{0}C\theta S\phi$$

(20) $$P_{4}=N_{0}C\theta C\phi$$

### 3.5. Inclination Calculation

There two solutions for DSRV trimming control. One is that the thrusters are used for trimming control. It is difficult to achieve the desired accuracy based on the thrusters alone, and the thrust allocation strategy will be very complex. Moreover, the thruster-based method is rather energy-consuming. As a result, this solution is restrained from widespread application. The other solution adopts load regulation, including the longitudinal transfer of ballast water, ballast iron or ballast lead. This is energy-saving and can meet the requirement of accuracy at the same time [63,64,65,66,67].

It is a challenge for DSRVs in heeling control. For higher precision in the heeling control of underwater docking operations, a pump-valve linkage system was adopted. The heeling angle is controlled by the change of the transverse position of the ballast water.

**Figure 8:**
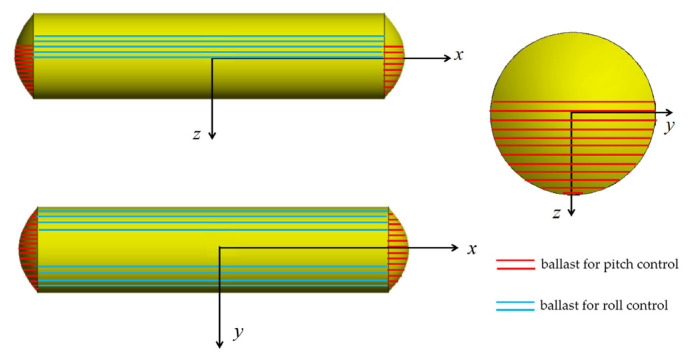
Layout of platform water ballasts.

There are six regulating water tanks designed in the platform as shown in Figure 8. Four are designed outside the pressure hull for heeling angle control with two on each side of the body. The other two are designed inside the pressure body for trimming control with one at the bow and the stern, respectively. Pumps and solenoid valves are equipped to engage in the transfer of the ballast water to achieve precise control of the trimming angle and heeling angle.

The PID control with intelligent integration is used to determine the opening and closing of the solenoid valves during the automatic trimming and heeling control. The method is shown in Equation (21).

(21) $$t_{j}(k)=K_{Pj}e_{j}(k)+K_{Ij}\sum_{n=o}^{k}e(n)+K_{Dj}{\dot{e}}_{j}(k)$$

In Equation (21), j=4, 5 means the roll and pitch directions. $t_{j}(k)$ is the opening or closing time of the pump and valve upon the kth control output. When $\left|t_{j}(k)\right|$ is greater than the preset threshold value, the act of opening or closing will be triggered. $e_{j}(k)$ and ${\dot{e}}_{j}(k)$ are the deviation and rate of deviation change of the trimming angle or heeling angle upon the kth control input. $\sum_{n=o}^{k}e(n)$ is the item of intelligent integration. It is introduced when $e_{j}(k)$ is less than the preset threshold value and otherwise not involved. This method can effectively deal with the integral saturation problem and reduce the overshoot while eliminating the steady-state error as well. $K_{Pj}$, $K_{Ij}$ and $K_{Dj}$ are the proportional, integral and differential regulating coefficients, respectively.

## 4. Numerical Simulations and Analysis

In order to verify the effectiveness of the proposed method, contrastive simulation experiments were carried out with the classic S-plane method. Water currents were included in the experiments to examine the robustness of the proposed control method against external impacts.

**Figure 9:**
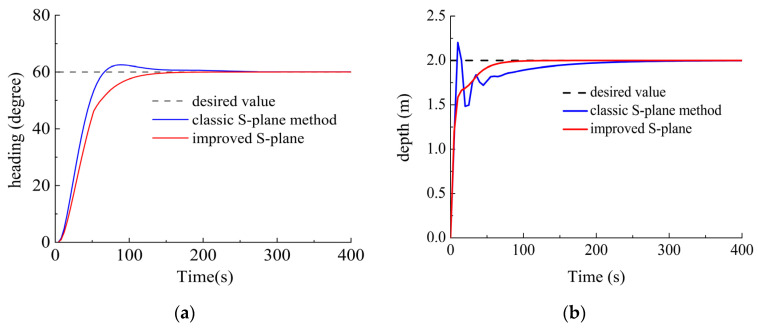
Contrastive control results (Scenario I). (**a**) Results of heading control; (**b**) results of depth control.

Scenario I. The DSRV was stationary on the water surface with the desired heading angle of 60° and desired depth of 2 m. The current was 0.2 m/s with a flow angle of 20°. The comparison simulation results are shown in Figure 9.

As shown in Figure 9a, the classic S-plane method showed a maximum overshoot of 2.8° with over 200 control beats before reaching the steady state. However, the improved S-plane method enabled the system to become steady in a stable way in a shorter time, with almost no overshoot or oscillation. 

As shown in Figure 9b, the maximum overshoot of the classic S-plane method was 0.32 m. Significant oscillations occurred during the 25–55th control beats. While the improved S-plane method reached the steady state more responsively without overshoot or oscillation.

**Figure 10:**
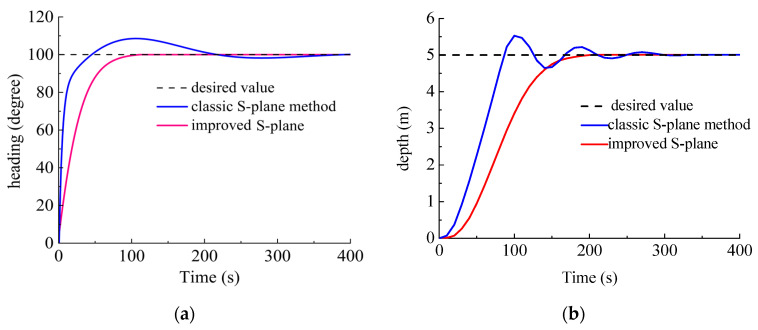
Contrastive control results (Scenario II). (**a**) Results of heading control; (**b**) results of depth control.

Scenario II. The DSRV was stationary on the water surface with the desired heading angle of 100° and desired depth of 5 m. The current was 0.5 m/s with a flow angle of 30°. The comparison simulation results are shown in Figure 10.

As shown in Figure 10a, faced with more challenging water conditions this time, the S-plane method showed a maximum overshoot of 8.7° and it took over 230 beats to reach steady. A striking contrast was made by the improved S-plane method, which smoothly stabilized the system with less energy consumed.

As shown in Figure 10b, the challenging water conditions hindered the classic S-plane method with a maximum overshoot of 0.49 m, with continuing oscillations during the 100–280th beats. In contrast, it took the improved S-plane method less than 200 beats to reach steady with much higher control accuracy.

Faced with the interference from water currents, the improved S-plane control method provided higher control accuracy and response with barely any overshoot or oscillation compared with the classic S-plane method. The simulation results proved the strong robustness of the proposed control method against current interference.

## 5. Results and Analysis of Pool Experiment

**Figure 11:**
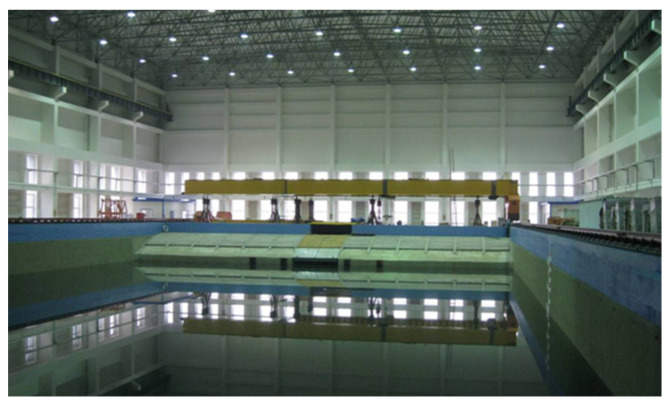
Site of pool experiment.

The pool experiment was carried out in a deep-water pool as shown in Figure 11. The pool has a wave maker that can generate water currents as in real seawater. The experiment was conducted to verify the feasibility of the DSRV control system.

**Figure 12:**
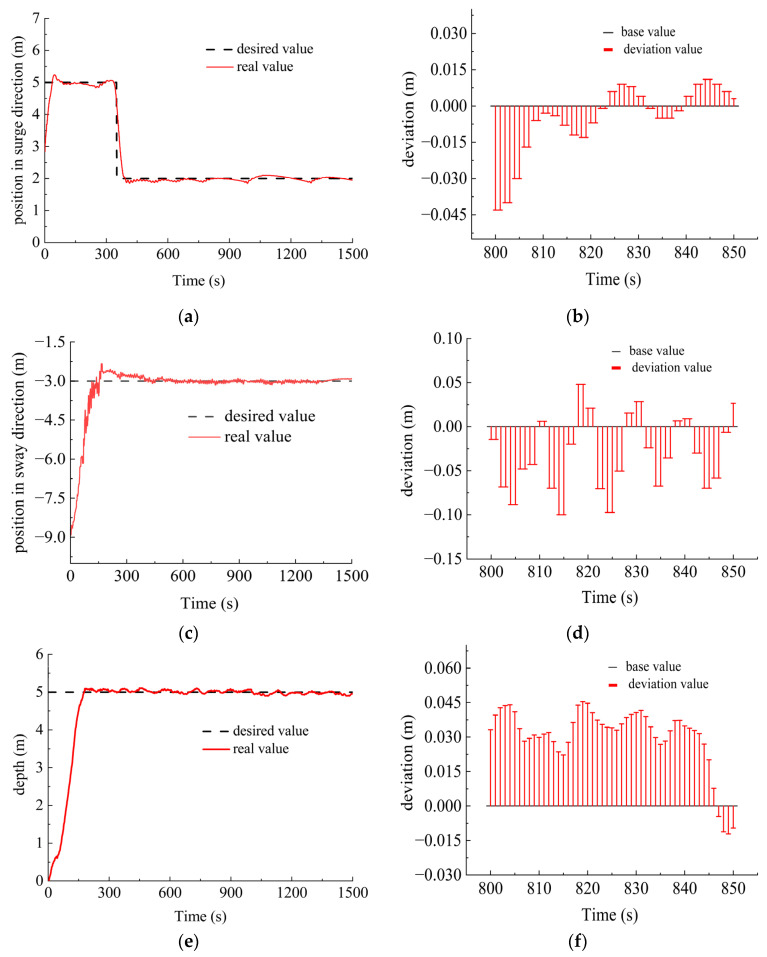

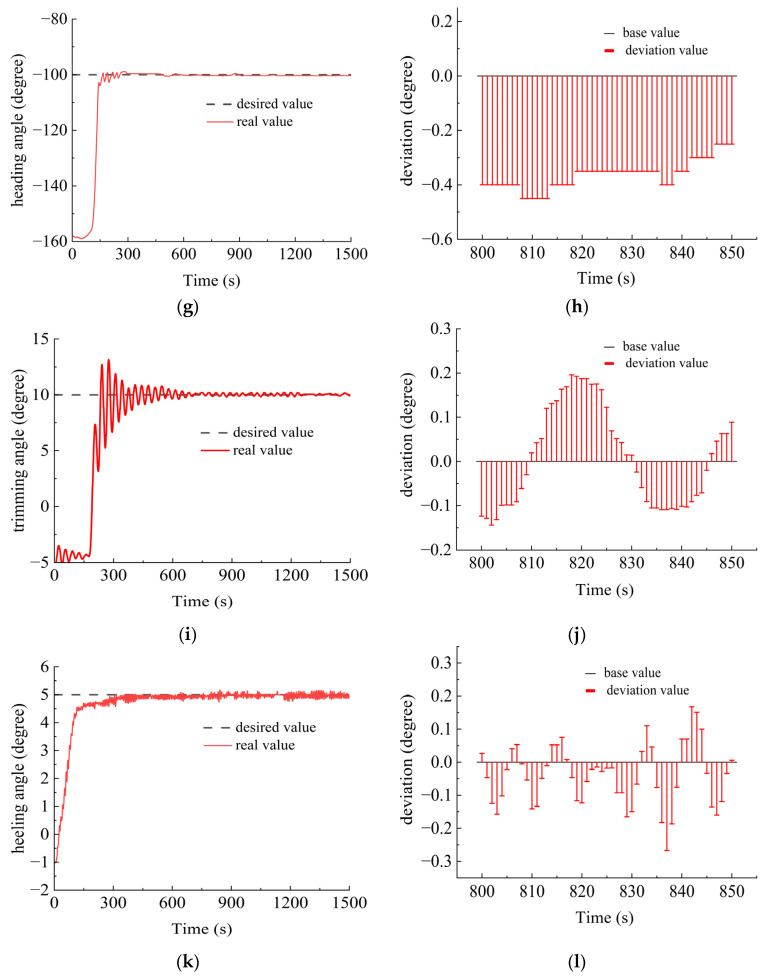
Control results of pool experiment. (**a**) Position in surge direction; (**b**) deviation of position in surge direction; (**c**) position in sway direction; (**d**) deviation of position in sway direction; (**e**) depth; (**f**) deviation of depth; (**g**) heading angle; (**h**) deviation of heading angle; (**i**) trimming angle; (**j**) deviation of trimming angle; (**k**) heeling angle; (**l**) deviation of heeling angle.

In the experiment, the platform was initially positioned at 2.8 m in the surge direction, −9 m in the sway direction, 0 m in the heave direction with a heading angle of −158°, a trimming angle of −5° and a heeling angle of −1°. The desired control targets were 5 m, 2 m in the surge direction, −3 m in the sway direction, 5 m in the heave direction with a heading angle of −100°, a trimming angle of 10° and a heeling angle of 5°. In order to examine the robustness of the platform, water interference was involved with the current velocity of 0.3 m/s and flow direction of 30°. The experiment results, together with the detailed deviation from the desired value ranging from 800–850 s during the control process are shown in Figure 12. 

As shown in Figure 12 during the 800–850 s when the system reached the steady state, the control deviation in the surge direction was within 0.05 m, that in the sway direction within 0.1 m and that in the heave direction within 0.05 m together with the heading deviation within 0.5°, trimming deviation within 0.2° and heeling deviation within 0.3°. The robustness and performance of the designed DSRV control system were well proved in the pool experiment.

## 6. Results and Analysis of Sea Trials

**Figure 13:**
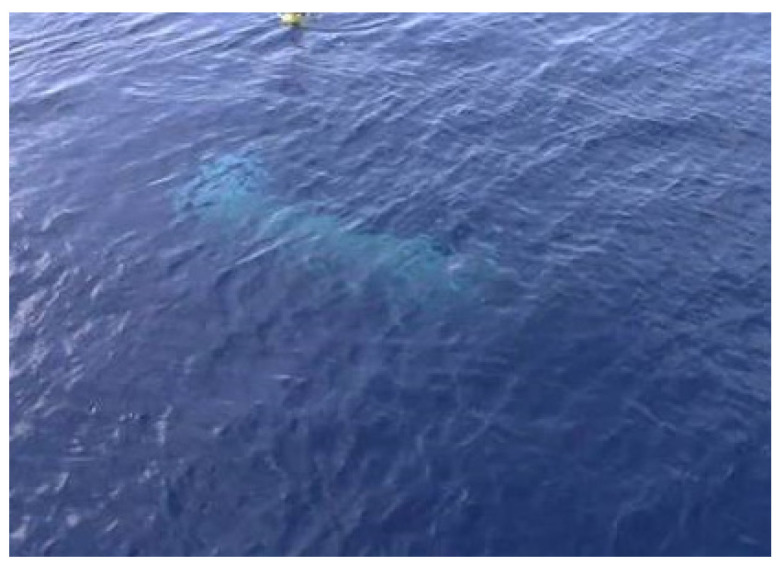
Environment of sea trials.

In order to verify the comprehensive performance of the DSRV control system, sea trials were also carried out. As shown in Figure 13, the sea trials proceeded in open waters with current interference. The motion control in six DOFs was conducted first, followed by the long-distance navigation.

### 6.1. Motion Control

**Figure 14:**
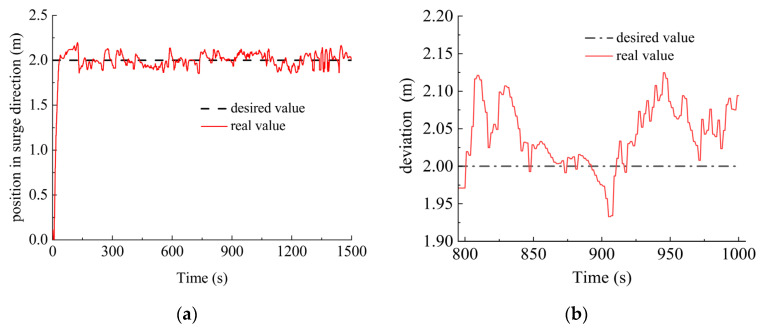

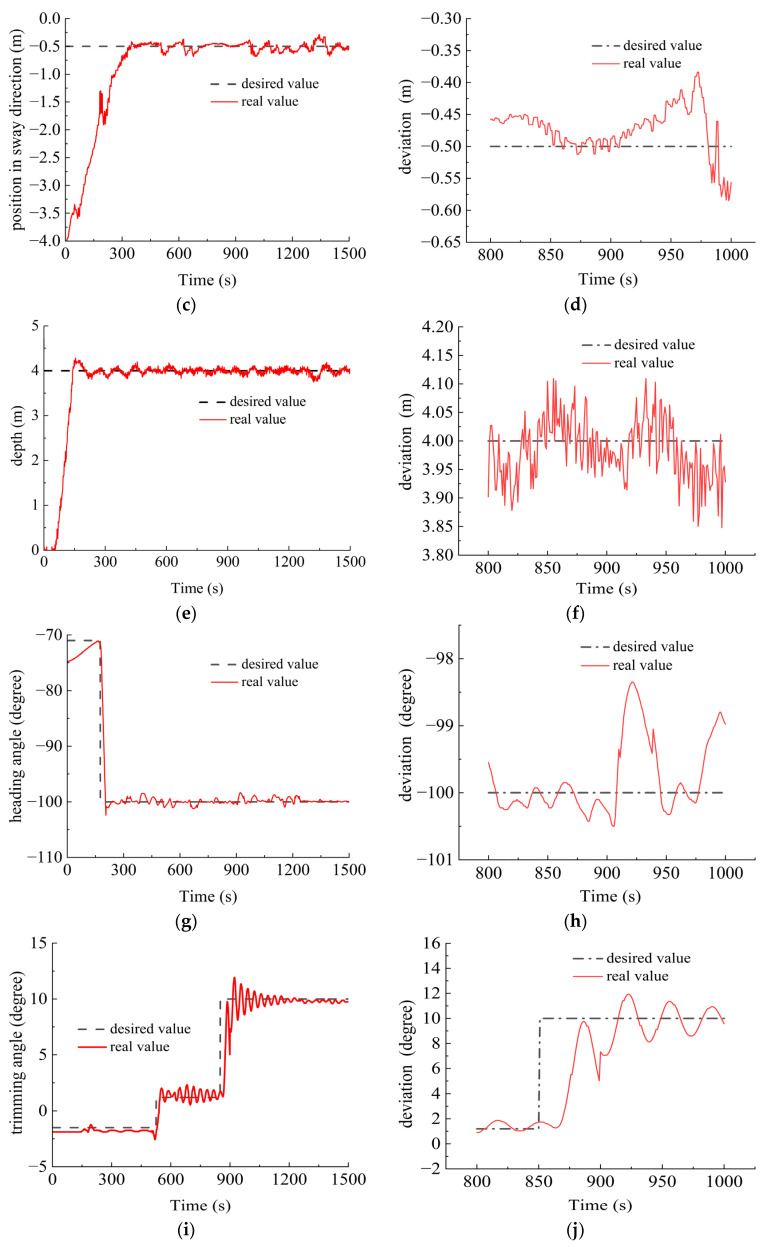

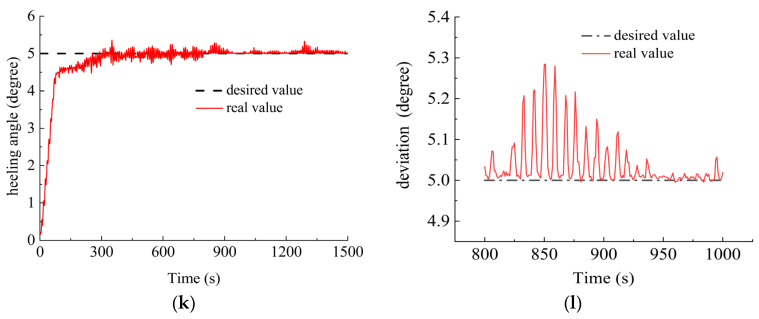
Results and detailed deviations of sea trial control. (**a**) Position in surge direction; (**b**) deviation of position in surge direction; (**c**) position in sway direction; (**d**) deviation of position in sway direction; (**e**) depth; (**f**) deviation of depth; (**g**) heading angle; (**h**) deviation of heading angle; (**i**) trimming angle; (**j**) deviation of trimming angle; (**k**) heeling angle; (**l**) deviation of heeling angle.

In the sea trial of motion control, the DSRV was initially positioned at 0 m in the surge direction, −4 m in the sway direction, 0 m in the heave direction with a heading angle of −76°, a trimming angle of −1.9° and a heeling angle of 0.3°. The desired control targets were 2 m in the surge direction, −0.5 m in the sway direction, 4 m in the heave direction with a heading angle of −100°, a trimming angle of −1.2°, 1.2°, 10° and a heeling angle of 5°. The sea trial results, together with the detailed deviation from the desired value ranging from 800–1000 s during the control process are shown in Figure 14.

As shown in Figure 14, after the system reached the steady state, the control deviations in the surge, sway and heave directions ranged within 0.2 m, with deviations of no more than 1° in heading, trimming and heeling angles control ranging from the 800–1000 s when the system reached the steady state. Despite the slight oscillations, the control accuracy can satisfy practical engineering tasks such as underwater docking operation. It took less than 5 min for the system to reach the desired state. In addition, as shown in Table 1, the maximum overshoot, standard deviation and arithmetic mean value of the control data ranging from the 800–1000 s during the control process also verified the responsiveness and accuracy of the DSRV control system.

### 6.2. Long-Distance Navigation

Long-distance navigation then proceeded to verify the reliability and operational stability of the DSRV control system. The approximately 5 km-long route was completed with an average velocity of 2.0 m/s at a depth of approximately 1.0 m. The hardware and software architectures of the DSRV functioned well throughout the navigation with no hardware failure or software crash.

**Figure 15:**
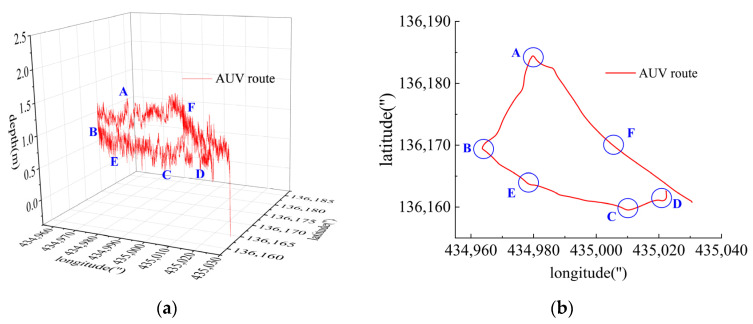
Long-distance route. (**a**) AUV route in three-dimension space; (**b**) AUV route in two-dimension plane.

As shown in Figure 15, there were sharp turns along the route, six of which were chosen (namely Region A–F) for detailed analysis of how the thrusters functioned in accordance with the thrust allocation strategy. Region A, Region B, Region C and Region D are provided with an analysis of the heading angle and thrusting force of the thrusters involved. Region E is provided with analysis of the heeling angle and thrusting force of the thrusters involved. Region F is provided with an analysis of the trimming angle and thrusting force of the thrusters involved.

**Figure 16:**
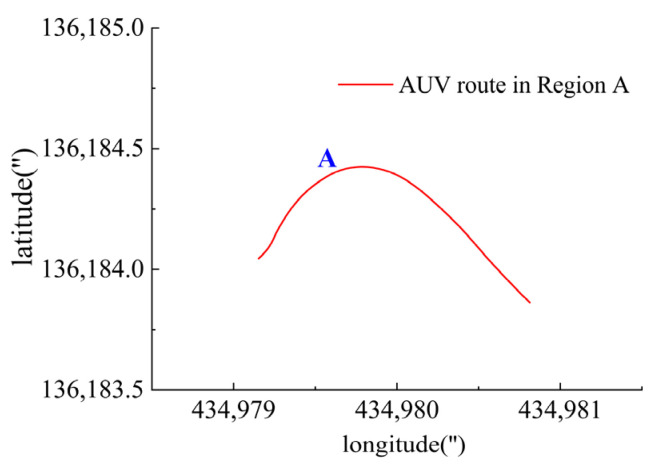
AUV route in Region A.

**Figure 17:**
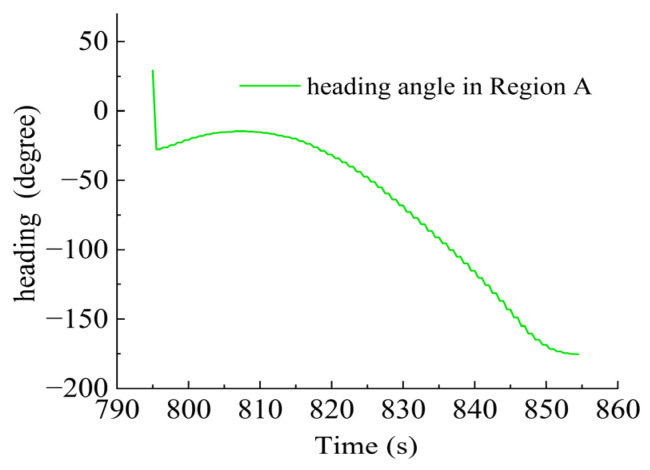
Heading angle in Region A.

**Figure 18:**
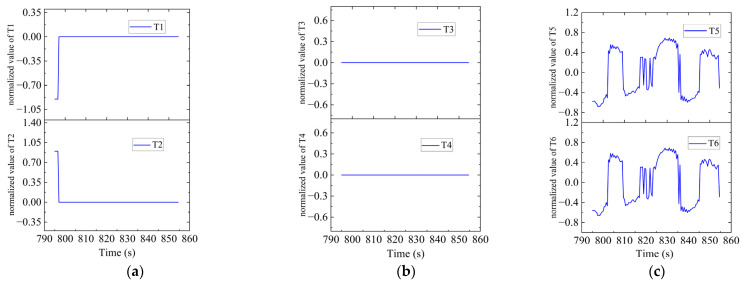
Thruster responses in Region A. (**a**) Response of T1 and T2; (**b**) response of T3 and T4; (**c**) response of T5 and T6.

In Region A, shown in Figure 16, T1, T2, T5 and T6 jointly contributed to the heading angle control as shown in Figure 17. As shown in Figure 18, around the turning point in Region A, T1 and T2 worked for a short period to facilitate the turning and then suspended working so that the freewheeling ensured smooth turning. T5 and T6 worked throughout this period for depth control. T3 and T4 were suspended during this period.

**Figure 19:**
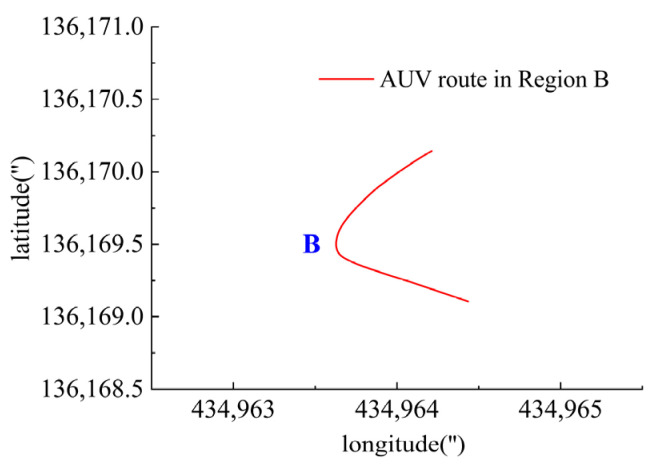
AUV route in Region B.

**Figure 20:**
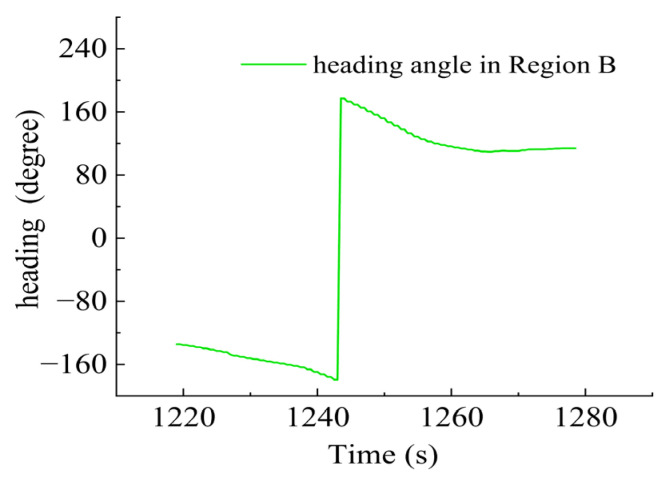
Heading angle in Region B.

**Figure 21:**
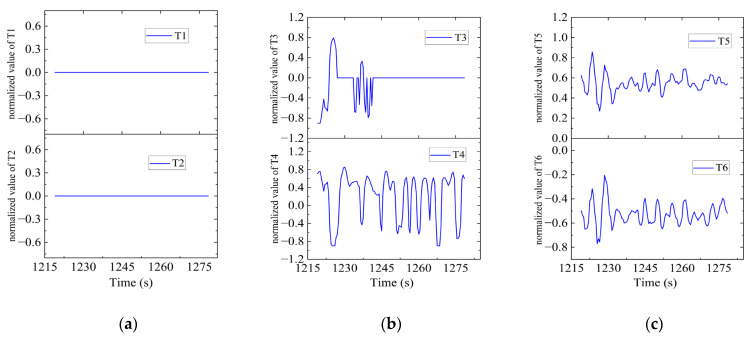
Thruster responses in Region B. (**a**) Response of T1 and T2; (**b**) response of T3 and T4; (**c**) response of T5 and T6.

In Region B, shown in Figure 19, T3, T4, T5 and T6 jointly contributed to the heading angle control as shown in Figure 20. As shown in Figure 21, around the turning point in Region B, T3 and T4 worked together for the heading angle control. At the same time, T5 and T6 worked for the depth control and prevented excessive heeling angles as well. T1 and T2 were suspended during this period.

**Figure 22:**
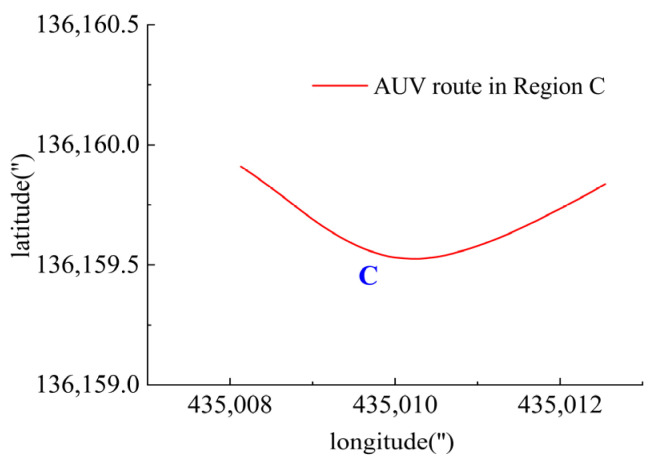
AUV route in Region C.

**Figure 23:**
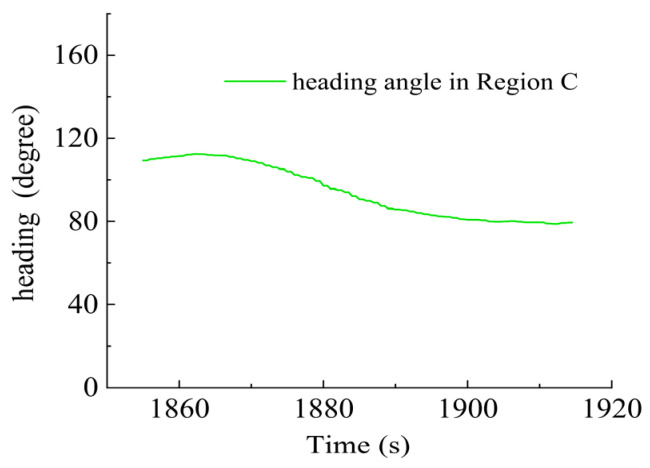
Heading angle in Region C.

**Figure 24:**
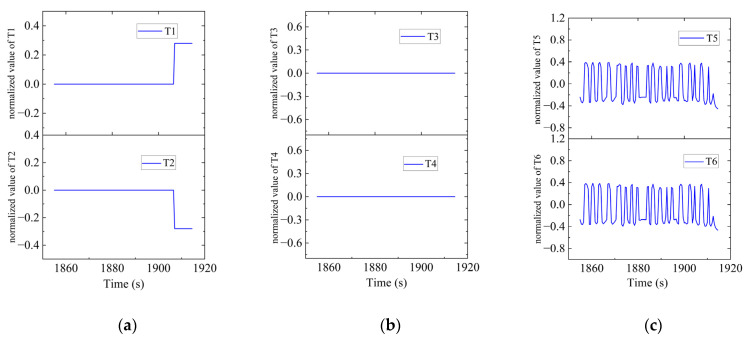
Thruster responses in Region C. (**a**) Response of T1 and T2; (**b**) response of T3 and T4; (**c**) response of T5 and T6.

In Region C, shown in Figure 22, T1, T2, T5 and T6 jointly contributed to the heading angle control as shown in Figure 23. As shown in Figure 24, around the turning point in Region C, T1 and T2 were suspended first to achieve stable turning and then restarted to complete the turning. T5 and T6 worked throughout this period for depth control against the water currents. T1 and T2 were suspended during this period.

**Figure 25:**
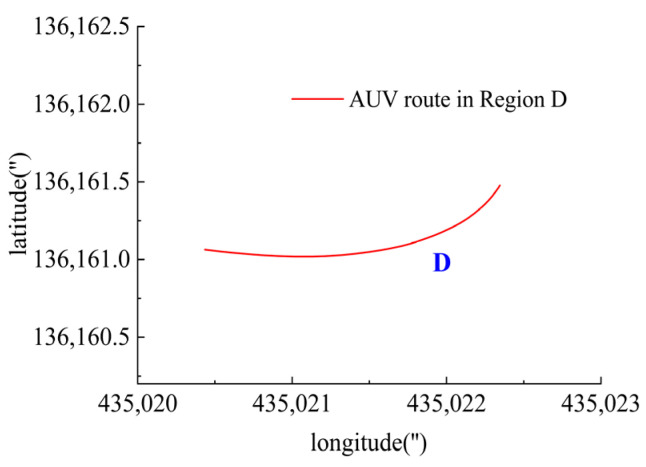
AUV route in Region D.

**Figure 26:**
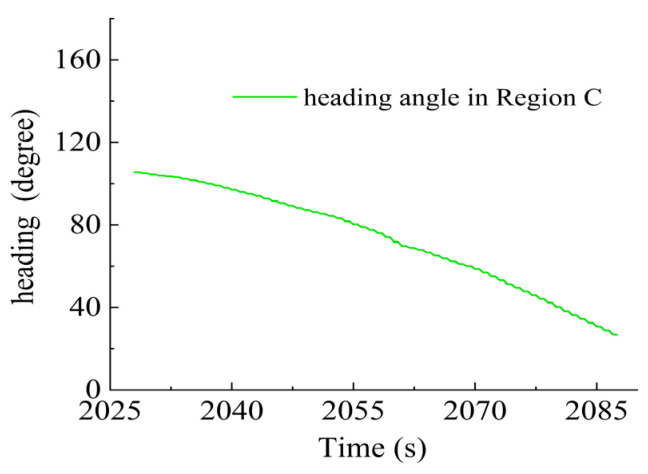
Heading angle in Region D.

**Figure 27:**
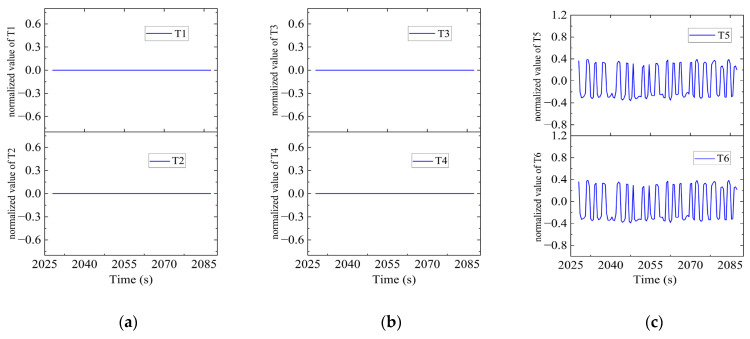
Thruster responses in Region D. (**a**) Response of T1 and T2; (**b**) response of T3 and T4; (**c**) response of T5 and T6.

In Region D, shown in Figure 25, T5 and T6 contributed to the heading angle control as shown in Figure 26. As shown in Figure 27, around the turning point in Region D, T1, T2, T3 and T4 were suspended to achieve stable turning since this was coming to the end of the route. However, T5 and T6 worked throughout this period for depth control against the water currents.

**Figure 28:**
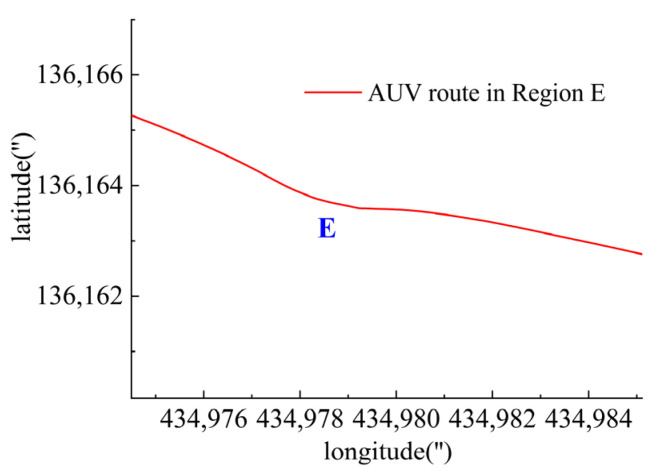
AUV route in Region E.

**Figure 29:**
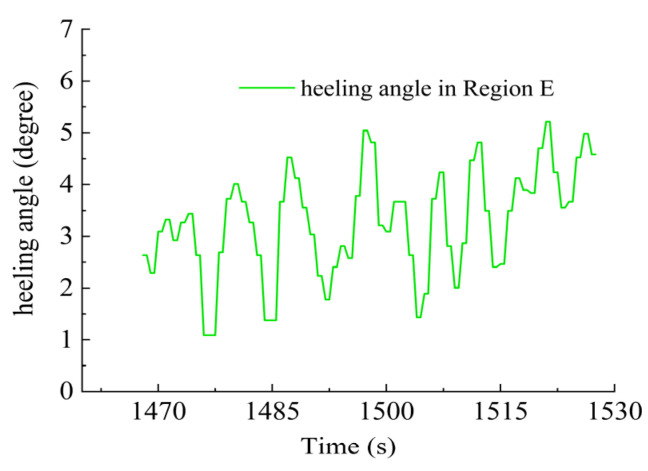
Heeling angle in Region E.

**Figure 30:**
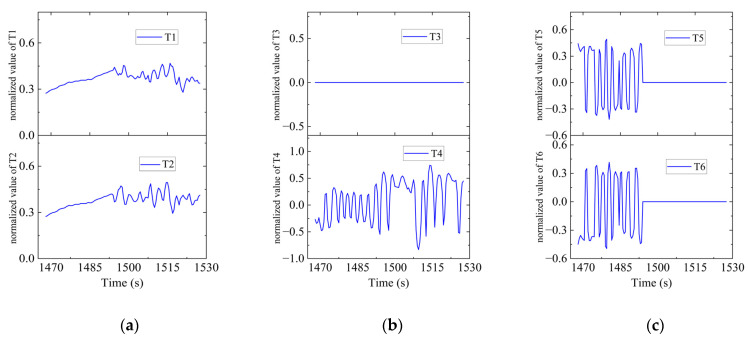
Thruster responses in Region E. (**a**) Response of T1 and T2; (**b**) response of T3 and T4; (**c**) response of T5 and T6.

In Region E, shown in Figure 28, T1, T2, T4, T5 and T6 jointly contributed to the heeling angle control as shown in Figure 29. As shown in Figure 30, T1 and T2 worked together for the velocity control. T3 was suspended and T4 worked for the heading control. At the same time, T5 and T6 worked to prevent excessive heeling angles. It can be seen that the heeling angle was controlled under 2.8° with an average of 1.5° during this period. In Region E, vertical thrusting forces restrained the heeling angle from overgrowing with the influence of water currents.

**Figure 31:**
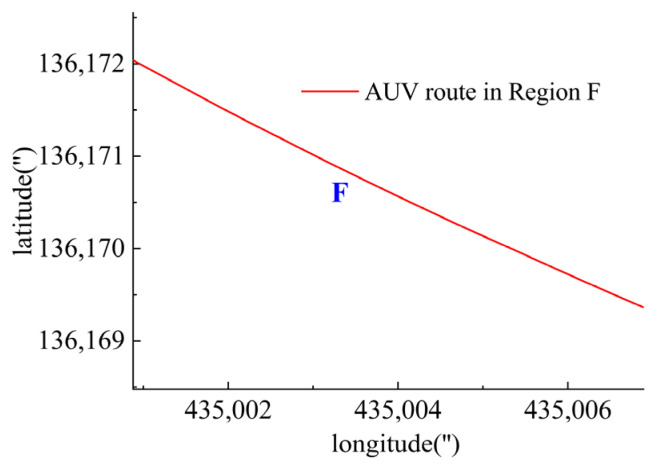
AUV route in Region F.

**Figure 32:**
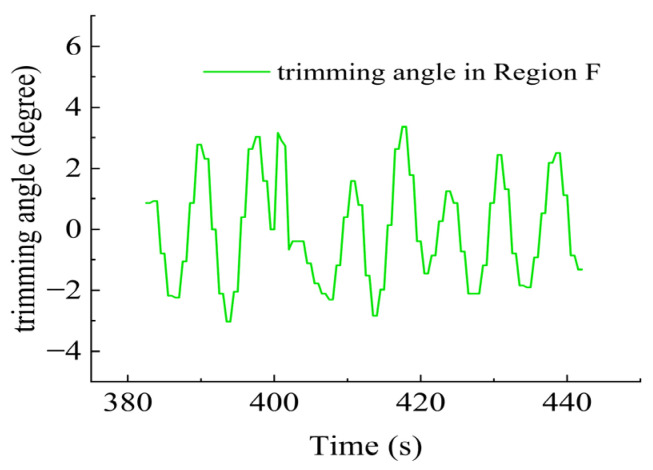
Trimming angle in Region F.

**Figure 33:**
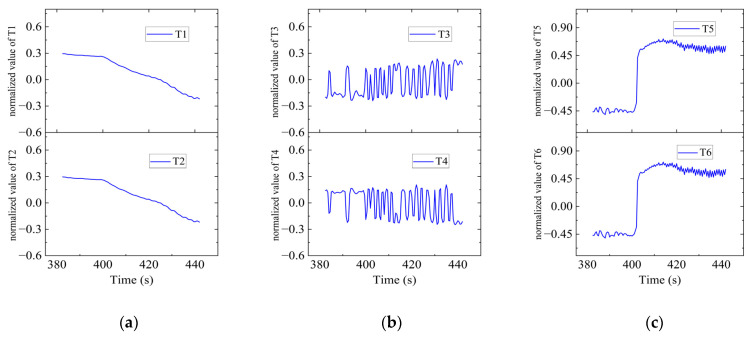
Thruster responses in Region F. (**a**) Response of T1 and T2; (**b**) response of T3 and T4; (**c**) response of T5 and T6.

In Region F, shown in Figure 31, all six thrusters worked since there was no requirement on the heading angle control, but the trimming angle data were provided as shown in Figure 32 due to the impact of water currents. As shown in Figure 33, T1 and T2 worked together for the velocity control. T3 and T4 were responsible for the expected heading angle. T5 and T6 worked to keep the DSRV at the desired depth.

## 7. Conclusions

In this study, a DSRV motion control system based on an improved S-plane method and intelligent integral PID is designed. The existing control methods are hindered from wide application as the result of complicated structures or demanding workloads in parameter regulation. Furthermore, the heeling angle is generally assumed to be insignificant and ignored by most of the existing control methods. As a result, practices of DSRV motion control in six DOFs are scarce due to the lack of actuators for heeling control.

In order to achieve precise DSRV motion control in six DOFs, the hardware architecture and software architecture of the platform were established. On this basis, the filter technology based on sparse representation was adopted to improve input accuracy. With the introduction of an adaptive control strategy and full consideration of the situational static load, an improved S-plane control method was put forward. The thrust allocation strategies were then studied in the case of control with and without inclination. A PID control with intelligent integration was also used to realize automatic inclination control.

The filtering technology was applied to control data of simulation experiments and sea trials, which improved the accuracy of inputs to the DSRV control system. Simulation experiments were carried out to compare the proposed control method with the classic S-plane control method to verify the superiority of the proposed control method. In the pool experiment of motion control in six DOFs considering the interference of water currents, the control accuracy was within 0.05 m in the surge direction, within 0.1 m in the sway direction, within 0.05 m in the heave direction, within 0.5° in the heading control, within 0.2° in the trimming control and within 0.3° in the heeling control. The control accuracy verified the robustness and good performance of the designed control system. In addition, sea trials were also carried out. In the sea trial of motion control in six DOFs, the control accuracy was within 0.2 m in directions of surge, sway and heave, within 1° in the control of heading angle and heeling angle as well as within 2° in the control of trimming angle. The control system reached the desired state within 5 min, which proved its effectiveness and responsiveness. Moreover, the 5km long navigation also verified the stability and reliability of the DSRV control system. It was verified that the six thrusters equipped functioned and responded in accordance with the thrust allocation strategy. In summary, the designed control system is stable and reliable with promising prospects in engineering practice especially for underwater docking operations.

The control method proposed in this paper focuses on the six-DOF motion control of AUVs with redundant thrusters and a pump-valve linkage system out of DSRVs’ needs for dynamic positioning. The feasibility and applicability of the proposed method on under-actuated underwater vehicles with rudder and fin are open to further discussions. In addition, with regard to the redundancy of the propulsion system of the platform, fault tolerance will be the focus of future studies to realize system reconstruction and fault tolerance in case of thruster failures.

## Figures and Tables

**Table 1:** Analysis of deviation in six DOFs (800–1000 s).

DOF	Initial Value	Desired Value	Maximum Overshoot	Standard Deviation	Arithmetic Mean Value
surge	0 m	2 m	0.125 m	0.042 m	0.042 m
sway	−4 m	−0.5 m	0.116 m	0.049 m	0.021 m
heave	0 m	4 m	0.109 m	0.053 m	−0.017 m
roll	0.3°	5°	0.284°	0.058°	0.037°
pitch	−2°	−1°, 1°, 10°	1.931°	2.843°	−1.341°
yaw	−76°	−100°	1.650°	0.559°	0.203°

## Data Availability

Not applicable.

## Abbreviations

**Abbreviation**	**Explanation**
DSRV	Deep Submergence Rescue Vehicle
DOF	Degree-of-Freedom
AUV	Autonomous Underwater Vehicle
PID	Proportional Integral Derivative
PD	Proportional Derivative
CPU	Central Processing Unit
DIO	Digital Input and Output
FOG	Fiber-Optic Gyroscope
DVL	Doppler Velocity Log
UDP	User Datagram Protocol
TCP	Transmission Control Protocol
K-SVD	K-Singular Value Decomposition
